# Impact of solar and wind development on conservation values in the Mojave Desert

**DOI:** 10.1371/journal.pone.0207678

**Published:** 2018-12-12

**Authors:** Sophie S. Parker, Brian S. Cohen, James Moore

**Affiliations:** 1 The Nature Conservancy, Los Angeles, California, United States of America; 2 The Nature Conservancy, San Diego, California, United States of America; 3 The Nature Conservancy, Las Vegas, Nevada, United States of America; Centro de Investigacion Cientifica y de Educacion Superior de Ensenada Division de Fisica Aplicada, MEXICO

## Abstract

In 2010, The Nature Conservancy completed the Mojave Desert Ecoregional Assessment, which characterizes conservation values across nearly 130,000 km^2^ of the desert Southwest. Since this assessment was completed, several renewable energy facilities have been built in the Mojave Desert, thereby changing the conservation value of these lands. We have completed a new analysis of land use to reassess the conservation value of lands in two locations in the Mojave Desert where renewable energy development has been most intense: Ivanpah Valley, and the Western Mojave. We found that 99 of our 2.59-km^2^ planning units were impacted by development such that they would now be categorized as having lower conservation value, and most of these downgrades in conservation value were due to solar and wind development. Solar development alone was responsible for a direct development footprint 86.79 km^2^: 25.81 km^2^ of this was primarily high conservation value Bureau of Land Management lands in the Ivanpah Valley, and 60.99 km^2^ was privately owned lands, mostly of lower conservation value, in the Western Mojave. Our analyses allow us to understand patterns in renewable energy development in the mostly rapidly changing regions of the Mojave Desert. Our analyses also provide a baseline that will allow us to assess the effectiveness of the Desert Renewable Energy Conservation Plan in preventing development on lands of high conservation value over the coming decades.

## Introduction

California has emerged as a world leader in the transition from fossil fuel use to renewable sources of electricity generation [1]. In 2015, California’s Clean Energy and Pollution Reduction Act (Senate Bill 350) codified the requirement that the state derive 50% of its electricity from renewable sources of energy by 2030 [2]. This followed the Federal Energy Policy Act (FEPA) of 2005, which called for the development of at least 10,000 megawatts of renewable energy generation on public lands by 2015. Due to these pieces of legislation, and other policy and economic incentives [1,2], solar and wind power generation in California has increased relatively rapidly (by 270%) since 2011 [3]. As of 2016, renewable energy facilities in California covered 400,000 km^2^ of land, more than in Germany and 188 other countries [4]. On some days, renewable sources of energy provide the majority of electricity to the state [5].

Most of California’s renewable energy comes from utility scale solar energy and large wind farms in the Mojave and Sonoran Deserts [2], which are regions recognized as having exceptional solar insolation and wind resource values [6]. However, California’s deserts are also one of the last great wilderness areas within the contiguous United States, containing thousands of square kilometers of intact, relatively undisturbed desert habitat [7]. Desert lands constitute 28% of California’s landmass and are known to contain 37% of its native plant taxa [8]. The desert southwest has been designated as a “hotspot” of endemism and endangered and threatened species occurrences [9], and desert systems are fragile and slow to recover once disturbed, due to their arid climate, delicate soils, and slow pace of ecological succession [7, 10].

Given the potential for conflict between renewable energy development and conservation in the California desert, The Nature Conservancy released the Mojave Desert Ecoregional Assessment (MDEA) [7] to help guide decision-making about the siting of renewable energy facilities not only in California’s portion of the Mojave, but also in the three adjacent states of Nevada, Utah, and Arizona. This analysis included a wall-to-wall assessment of conservation values in the four-state ecoregion. In subsequent years, increasing attention has been paid to documenting the known and potential environmental impacts of utility scale solar energy and wind development [11,12,13,14,15,16,17,18], and a handful of studies have been released to help guide siting of utility scale solar energy and wind farms to locations in the California deserts where there are fewer conflicts with other land uses [19,20,21]. These efforts have occurred in concert with the development of the Desert Renewable Energy Conservation Plan (DRECP), which culminated with the release of a Land Use Plan Amendment (LUPA) for California’s Bureau of Land Management (BLM) desert lands in the fall of 2016. The DRECP LUPA designates 1,570 km^2^ of BLM lands as development focus areas for solar, wind, and geothermal facilities. To conserve biological, cultural and other values, the DRECP LUPA also designates 17,000 km^2^ of BLM lands as National Conservation Lands, Areas of Critical Environmental Concern, wildlife allocations, and National Scenic and Historic Trail management corridors [22]. In early 2018, the Bureau of Land Management began gathering public feedback on potential amendments to the DRECP, and the conservation management actions laid out in the current land use plan may eventually be altered.

During the decade between the passage of the FEPA of 2005 and the release of the DRECP LUPA in 2016, applications for the development of utility scale solar energy and wind facilities were reviewed and approved by the appropriate agencies on a case-by-case basis. A total of 53 utility scale solar energy facilities and roughly 1,000 wind turbines were built or expanded in the California deserts during this time (Table 1). Some of these “pre-DRECP legacy projects” have significant, documented conservation impacts [4]. They also represent California’s decision-making regarding utility scale solar energy and wind facility siting prior to the release of the DRECP LUPA’s designated development focus areas, which are meant to be priority locations for the development of renewable energy facilities in the California deserts.

**Table 1:** Recent renewable energy development in the California Mojave Desert.

Year	Number of Installations	Maximum MegaWatts of Capacity
2009	2	180
2010	1	21
2012	1	29
2013	16	2,078
2014	11	1,132
2015	6	407
2016	16	1,385
**Total**	**53**	**5,231**

Data compiled from ABB-Ventyx. 2017. e_plants_point; 2017 [cited 10 April 2018] Atlanta, GA: ABB-Ventyx.

Renewable energy facilities are likely to continue to be built in California’s deserts, given the state’s growing population and increasing energy demands [23] as well as the likelihood that the state legislature will enact increasingly ambitious greenhouse gas emission goals. With the DRECP LUPA in place, the location and pattern of utility scale solar energy and wind development over the next decades may change. To gauge the success of the DRECP as a land use policy intended to enable better protection of conservation values in the California deserts, while also allowing renewable energy development to occur on lands of less ecological importance, we must first have some understanding of how pre-DRECP legacy projects impacted conservation values.

The MDEA includes a map of conservation values in the California Mojave Desert that was developed using satellite imagery from 2009 and 2010, after the FEPA was passed in 2005. Here we use the same decision-rules employed in the generation of the MDEA map to reassess conservation values in the Mojave Desert using up-to-date satellite imagery that depicts pre-DRECP legacy projects built between 2009 and 2017. We focused our efforts on two subregions of the Mojave Desert where projects were built, but that differed in terms of the percentage of public lands they contain, as the FEPA of 2005 specifically focused on siting large renewable energy facilities on public lands. The purpose of our analyses was to determine if the rush to develop renewable energy prior to the release of the DRECP was associated with changes in public or private land use detectable through examination of satellite imagery, and how these observable changes would alter our remote assessment of the conservation value of lands in the Mojave Desert. Our analysis not only provides an up-to-date assessment of the conservation value of these lands prior to the passage of the DRECP, therefore allowing for future reassessments against a pre-DRECP baseline, but it also provides an understanding of how land use in the Mojave Desert changed after the FEPA of 2005 was enacted.

## Methods

**Fig 1:**
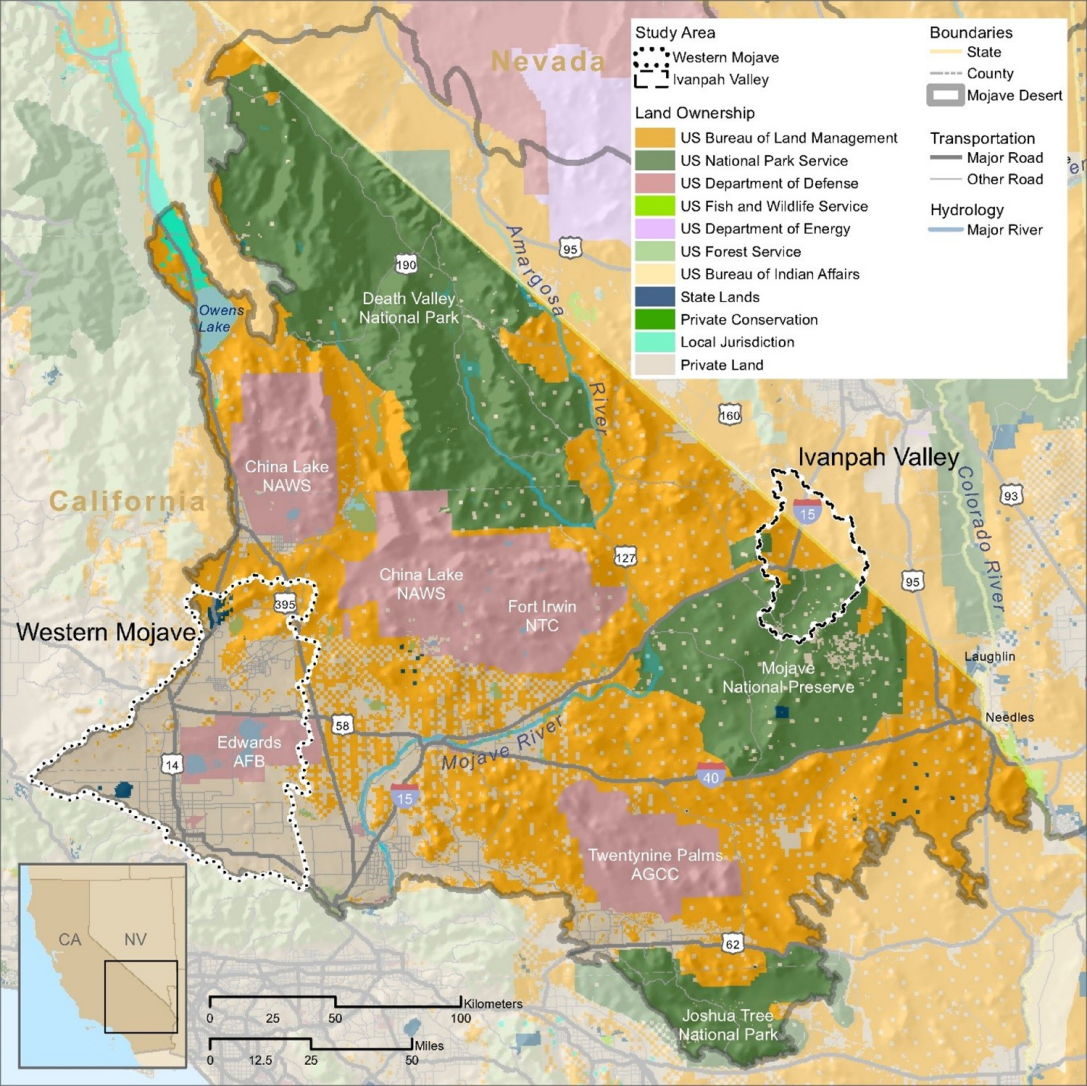
Land ownership in the California Desert. The Study Areas are shown in the Western Mojave and Ivanpah Valley are shown with dotted and dashed outline, respectively.

We selected two study areas within the California portion of the Mojave Desert Ecoregion in which to focus our analysis: the Ivanpah Valley and the Western Mojave. We used watershed boundaries to delineate these two study areas [24]. Because one of our intentions was to assess pre-DRECP development impacts, we chose study areas in the California portion of the Mojave Desert, where the DRECP applies. However, because watershed boundaries fall across state lines, a portion of the Ivanpah Valley study area is in the state of Nevada. Because a section of the FEPA of 2005 was specifically focused on siting large renewable energy facilities on public lands, we selected two study areas that reflect differences in land ownership throughout the California Mojave Desert: the Western Mojave Desert, which consists mostly of privately owned lands, and the Ivanpah Valley, which consists primarily of publicly owned lands (Fig 1). The majority of the publicly owned lands are managed by the BLM. In addition, each of the two study areas includes one of the 10 “evolutionary hotspots” identified in the Mojave Desert Ecoregion, where intraspecific genetic divergence and diversity were high [25]. Most lands within the California Mojave (80%) are categorically excluded from utility scale solar energy and large wind farm development because they are managed by the National Park Service, the Department of Defense, or are otherwise off-limits for development. Therefore, we viewed point data [26] depicting the locations of renewable energy projects throughout the Mojave Desert to ensure that our study areas contained several projects.

Our analysis involved remote assessment of current conservation values for all lands within in our two study areas, and comparison of the current conservation values with those reported in the MDEA [7] to understand how conservation values have changed over time. We used the same hexagonally-shaped planning units (HPUs) used in the MDEA; each HPU covers 2.59 km^2^ of desert lands, equivalent to a square mile—a size that allows for easy comprehension by decision-makers.

The original assessment of conservation values in the MDEA was conducted based on the principles of systematic conservation planning [27] and is described in detail in Appendix A of the MDEA [7]. Briefly, we synthesized spatially-explicit information on a suite of conservation targets--including 521 species, 44 ecological systems, and all seeps and springs--and anthropogenic disturbance found in each HPU, and then used MARXAN software to identify the relative value of each HPU in meeting our conservation goals, and to preliminarily assign each HPU to one of four conservation value categories (Table 2). Post-MARXAN processing was used to validate and correct these assignments; three scientists with desert land use expertise remotely inspected current imagery and employed predetermined decision rules to gauge whether each HPU had been appropriately assigned. Corrections were made as appropriate. All Post-MARXAN processing done remotely, via phone and computer screen sharing to allow each observer to provide unbiased review.

**Table 2:** Definition of conservation value categories.

Category	Explanation
Ecologically Core (Dark Green)	• Highest conservation value • Largely undisturbed and unfragmented • Support conservation targets • Identified as critical to fully protect for the long-term conservation of the ecoregion’s biological diversity • Their conservation value is highly dependent on connections with Ecologically Intact and Moderately Degraded lands around them
Ecologically Intact (Light Green)	• Relatively undisturbed and unfragmented • Support conservation targets • Require levels of protection that will allow them to remain relatively undisturbed and to continue to support ecological processes and provide habitat and habitat connectivity for native animals, plants, and communities • Most are functionally equivalent to Ecologically Core lands and may contain many of the same conservation targets, including sensitive species • May support more widespread ecological systems (e.g., creosote-scrub) that have lower conservation goals
Moderately Degraded (Orange)	• Lands with roads or OHV trails, or near urban, agricultural, or other developments • Partially to moderately compromised by fragmentation and other human impacts such as rural development, agriculture, OHV use, and military use. • Often maintain ecological functionality (e.g., maintain groundwater infiltration and flow, serve as sand sources, provide connectivity), provide habitat for native species, or are known to have conservation target occurrences • Their potential to provide long-term conservation value and to be restored is greater where they are located adjacent to Ecologically Intact lands
Highly Converted (Red)	• Lowest conservation value • Urban, agricultural and suburban lands that are heavily altered • Some support important conservation targets but the ecological context of these is compromised. • Some conservation targets, such as Burrowing Owls, migratory birds, and bats use or congregate in these heavily modified landscapes • These lands subsidize predatory species such as coyotes and ravens that can have detrimental effects on conservation targets such as the desert tortoise

Color code is used in maps to characterize lands in both the Mojave Desert Ecoregional Assessment (MDEA) and in this analysis.

In the current analysis, we updated the assessment of conservation values within our two study areas using the same post-MARXAN procedures used in the MDEA. We utilized ocular estimation [28] of land use change by the three expert observers to determine how much of each HPU inspected had changed. When the amount of land use change detected was close to the thresholds used in the decision rules (Table 3), we validated our estimations using the area measure tool in Esri ArcMap. The observers involved in this analysis were the same three individuals who performed the processing for the MDEA as described above. The same predetermined decision rules used in the MDEA were used in this analysis (Table 3).

**Table 3:** Decision rules for conservation value assignment.

Designation in 2010 MDEA	Designation in Current Analysis	Reason
Ecologically Core	Ecologically Core	no change
Ecologically Core	Ecologically Intact	An Ecologically Core HPU^1^ was downgraded to Ecologically Intact if it was not adjacent to another Ecologically Core HPU and was adjacent to: at least 4 Moderately Degraded HPUs OR one Highly Converted and two Moderately Degraded HPUs OR two Highly Converted HPUs
Ecologically Core	Moderately Degraded	25% of land within the HPU was newly disturbed or converted OR 3 or more turbines were within the HPU^2^
Ecologically Core	Highly Converted	50% or more of land within the HPU was newly disturbed or converted
Ecologically Intact	Ecologically Intact	no change
Ecologically Intact	Moderately Degraded	25% of land within the HPU was newly disturbed or converted
Ecologically Intact	Highly Converted	50% or more of land within the HPU was newly disturbed or converted
Moderately Degraded	Ecologically Intact	The HPU was adjacent to and within 0.8 km^3^ of Interstate 15 and known tortoise fencing had occurred since 2010
Moderately Degraded	Moderately Degraded	no change
Moderately Degraded	Highly Converted	50% or more of land within the HPU was newly disturbed or converted
Highly Converted	Highly Converted	no change

^1^ HPU: hexagonally-shaped planning unit, measuring approximately 2.59 km^2^ (equivalent to one square mile).

^2^ This decision rule was used in the assignment of conservation values for the Mojave Desert Ecoregional Assessment (MDEA) but was not specified in the text of that document. Locations with 3 or more turbines per HPU were estimated to have 25% of the HPU impacted by the turbines and associated roads, as determined by ocular estimation.

^3^This value corresponds to field research suggesting a dead-zone effect of unfenced roads out to a distance of 1.6 km on high-traffic roads.

The imagery used for our analysis included 2009 NAIP for checking the condition of lands as they appeared during the generation of the 2010 MDEA, and 2014 NAIP, 2016 NAIP, and 2017 Landsat 8, along with various imagery dates and sources from current Esri ArcGIS Online Imagery Service, to assess the current 2017 conditions of lands. Given the time required for new renewable energy project approvals, any development that look place during the period of time after the release of the DRECP in September of 2016 and prior to capture of the 2017 Landsat 8 imagery used in our analysis, would have been the result of a project that had entered the approvals process prior to the release of the DRECP, and was therefore not subject to the DRECP LUPA. To compare the 2009 imagery to the “current” condition, we overlaid the boundaries of the HPUs used in the MDEA on top of the imagery for the “current” condition. We then determined, using our decision rules, whether there had been a change in the designation of conservation value for each HPU based on visible land use impacts.

Changes in designation that resulted in a lower conservation value we termed “downgrades”, and those that resulted in higher conservation value were “upgrades”. The reason for the new designation was recorded for each planning unit (Table 4).

**Table 4:** Categories of downgrades for each hexagonally-shaped planning unit (HPU).

Study Area	Designation in 2010 MDEA^1^	Reason for Downgrade	# HPUs
Ivanpah Valley	Ecologically Core	Adjacent to Moderately Degraded and Highly Converted HPUs^2^	2
		Solar Power Tower Development (new)	9
		Solar Photovoltaic Development (new)	5
	Ecologically Intact	Off Highway Vehicle Tracks Associated with Adjacent Development	1
		Solar Photovoltaic Development (new)	5
	Moderately Degraded	Solar Photovoltaic Development (new)	1
Western Mojave	Ecologically Core	Adjacent to Moderately Degraded and Highly Converted HPUs	2
		Agriculture Intensification	3
		Former Agriculture Converted to Intensive Agriculture	4
		Mine Expansion	2
		Sewage Treatment Expansion	1
		Wind Development (new)	2
	Ecologically Intact	Solar Photovoltaic Development (new)	9
		Wind and Solar Photovoltaic Development (contiguous development with new technology)	1
		Wind Development (contiguous development with new technology)	10
		Wind Development (new)	18
	Moderately Degraded	Agriculture Expansion	1
		Agriculture Intensification	1
		Mine Expansion	1
		Solar Photovoltaic Development (new)	9
		Solar Photovoltaic Development (new on agricultural lands)	11
		Wind and Solar Photovoltaic Development (new)	1
Grand Total			99

^1^ MDEA: 2010 Mojave Desert Ecoregional Assessment.

^2^ HPU: hexagonally-shaped planning unit, measuring approximately 2.59 km^2^ (equivalent to one square mile).

Agricultural Intensification differs from Agricultural Expansion in that Intensification is visible in satellite and aerial imagery as more intense ground disturbance associated with agriculture in an area that was defined as agricultural in the past, whereas Expansion involves the conversion of what was previously non-agricultural lands into agriculture.

Some HPUs containing mining, military, OHV, human communities, or waste disposal sites, if designated as Highly Converted, were buffered all around with Moderately Degraded lands because of the high likelihood of impacts from these types of land uses spreading to adjacent lands (i.e. trash blowing around, dust and trails from OHV racing activity, feral cats and dogs and weeds dispersing from human communities). Some mining lands are limited or contained by the topography of the adjacent landscape, and were therefore not subject to this rule. For developments that are highly planned, fenced, and contained (such as solar development) and where impacts are less likely to expand to adjacent lands, Highly Converted lands can be immediately adjacent to Ecological Core or Ecologically Intact Lands.

To reflect the change in conservation values due to known locations where tortoise fencing had been installed along Interstate 15, we decided that, if the Interstate intersected the HPU, it would remain as Moderately Degraded, even if it was fenced to exclude desert tortoise because it still represents unusable habitat. In cases where the HPU was adjacent to or within a 0.8-km highway buffer, if fenced it was changed from Moderately Degraded to Ecologically Intact. It is important to note that lands that had been upgraded due to tortoise fencing were also subject to being downgraded because of new land disturbance.

Given that wind turbines have three-dimensional aerial impacts on wildlife [29] despite their relatively small terrestrial footprint, we assigned all HPUs containing at least three large modern turbines to the Moderately Degraded category.

In addition to assessing changes in conservation value due to both solar and wind development, we completed an analysis of the land ownership and management where solar developments took place by developing a spatial database of the footprints of solar energy facilities and overlaying that database with a land ownership database. We developed our solar energy facilities database in Esri ArcMap by creating polygons delineating the solar installations visible on various image services, such as NAIP 2016 and Landsat 8 PanSharpened and storing these polygons in a file geodatabase. We then ran the Esri *Intersect* function with the solar facilities footprints and PADUSv1.4 [30] to identify the land owner and manager coincident with the solar installations.

## Results

**Fig 2:**
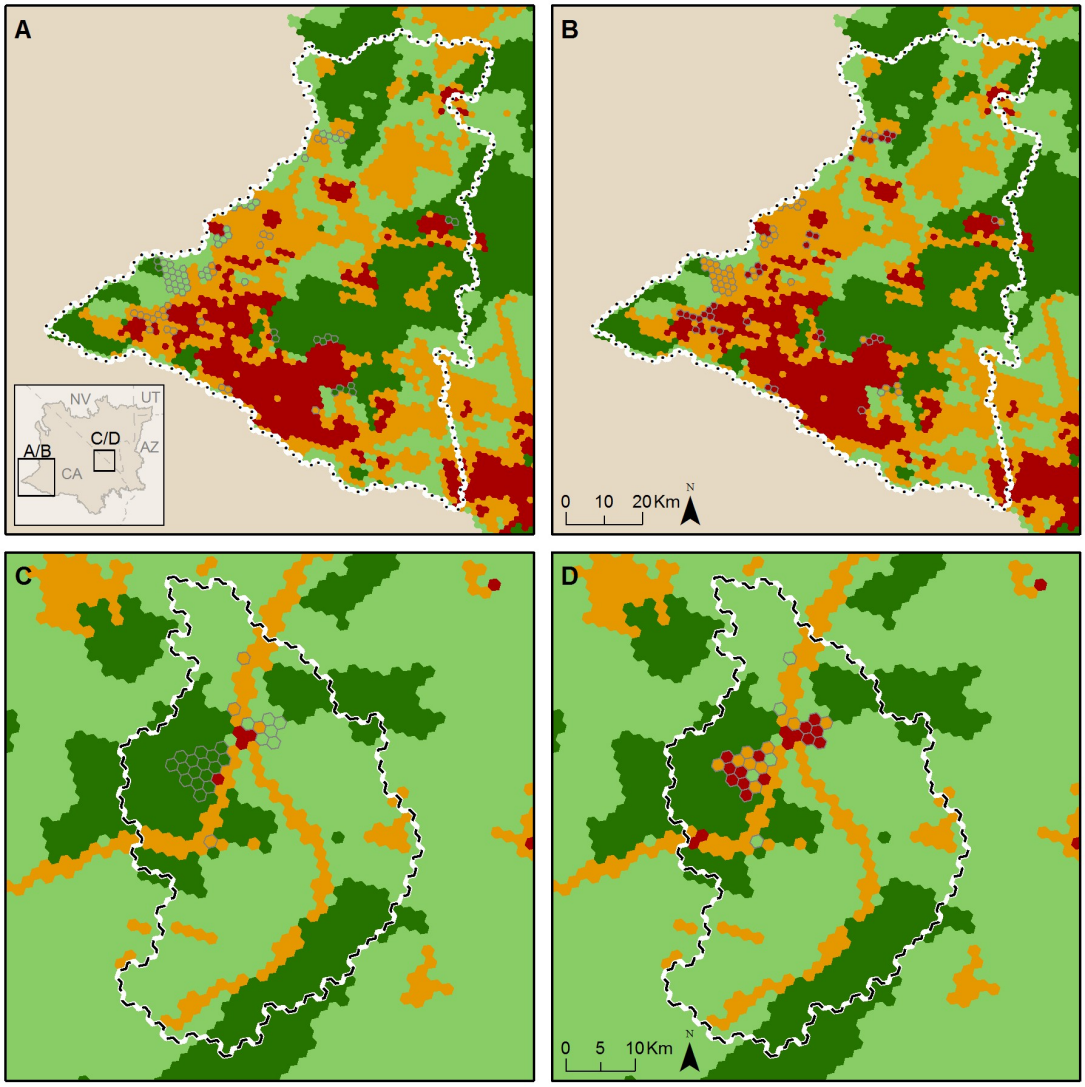
Comparison of conservation values. Conservation values are shown for 2009 (A and C) and 2017 (B and D) in the Western Mojave (A and B) and Ivanpah Valley (C and D) Study areas. The four colors shown on the maps (dark green, light green, orange, and red) correspond with the conservation value categories defined in Table 2.

**Fig 3:**
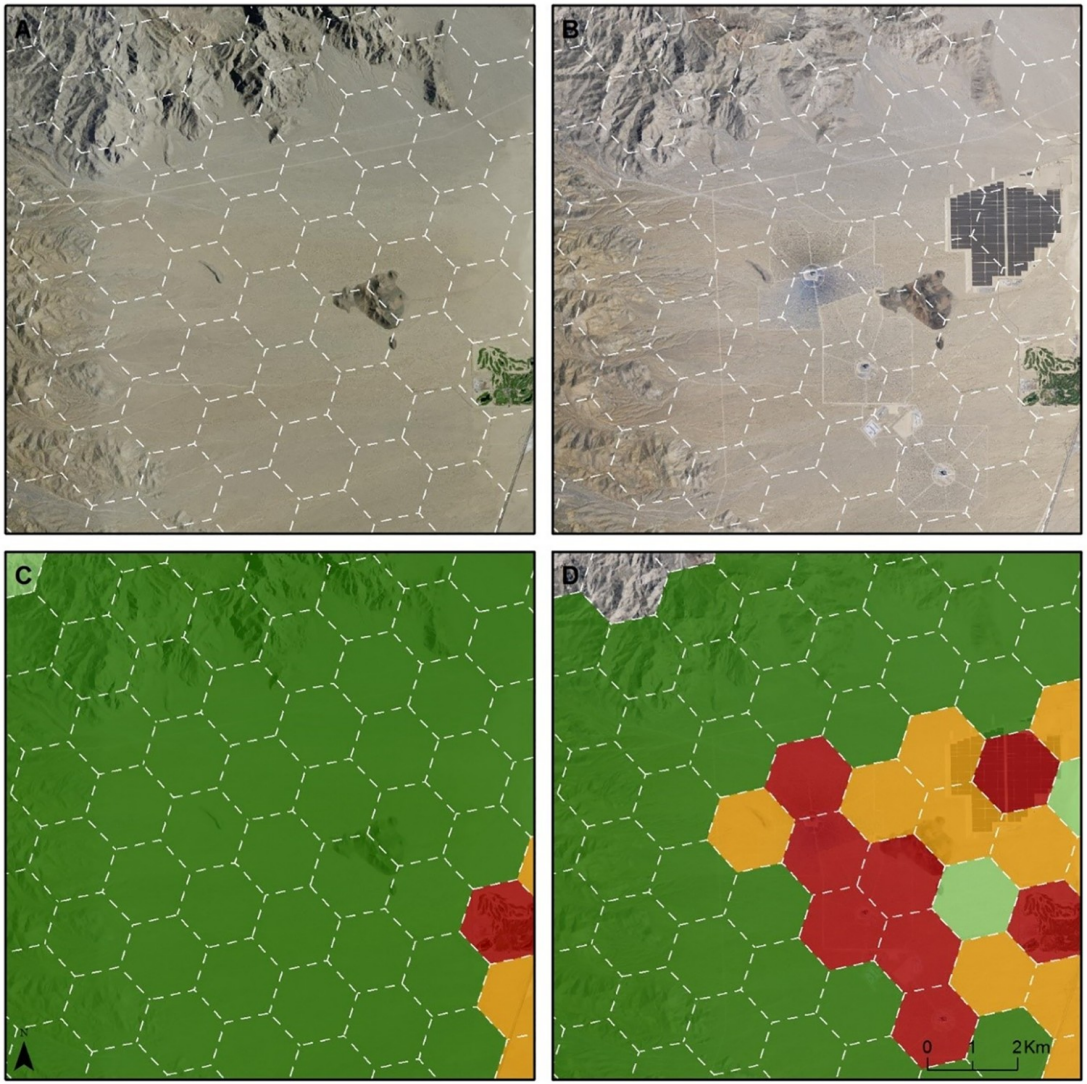
One Example of change in conservation value in Ivanpah Valley Due to solar energy development. A: 2009 imagery; B: 2016 imagery; C: Conservation Value in 2009; D: Conservation Value in 2017. The four colors shown on maps C and D (dark green, light green, orange, and red) correspond with the conservation value categories defined in Table 2.

**Fig 4:**
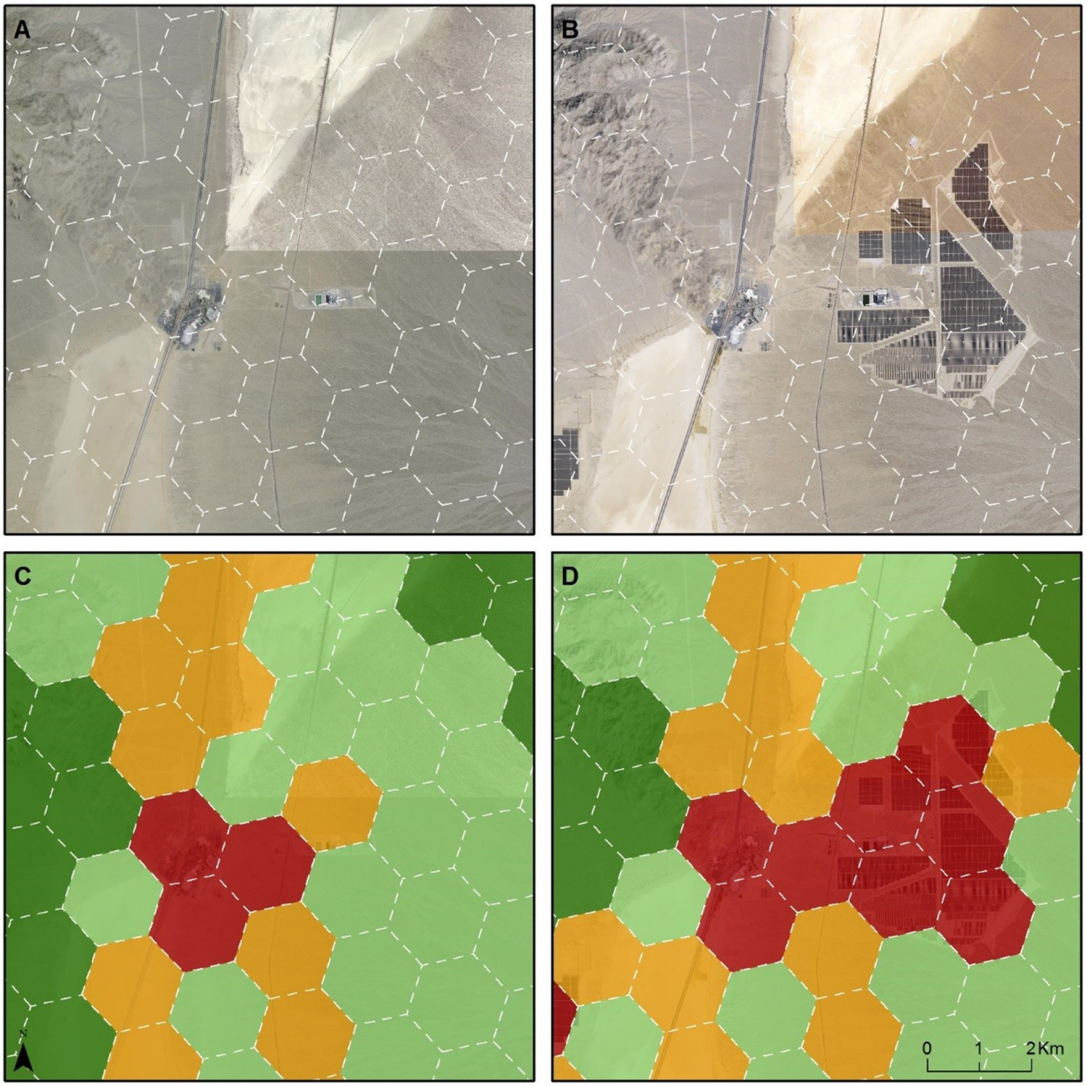
A second example of change in conservation value in Ivanpah Valley Due to solar energy development. A: 2009 imagery; B: 2016 imagery; C: Conservation Value in 2009; D: Conservation Value in 2017. The four colors shown on maps C and D (dark green, light green, orange, and red) correspond with the conservation value categories defined in Table 2.

**Fig 5:**
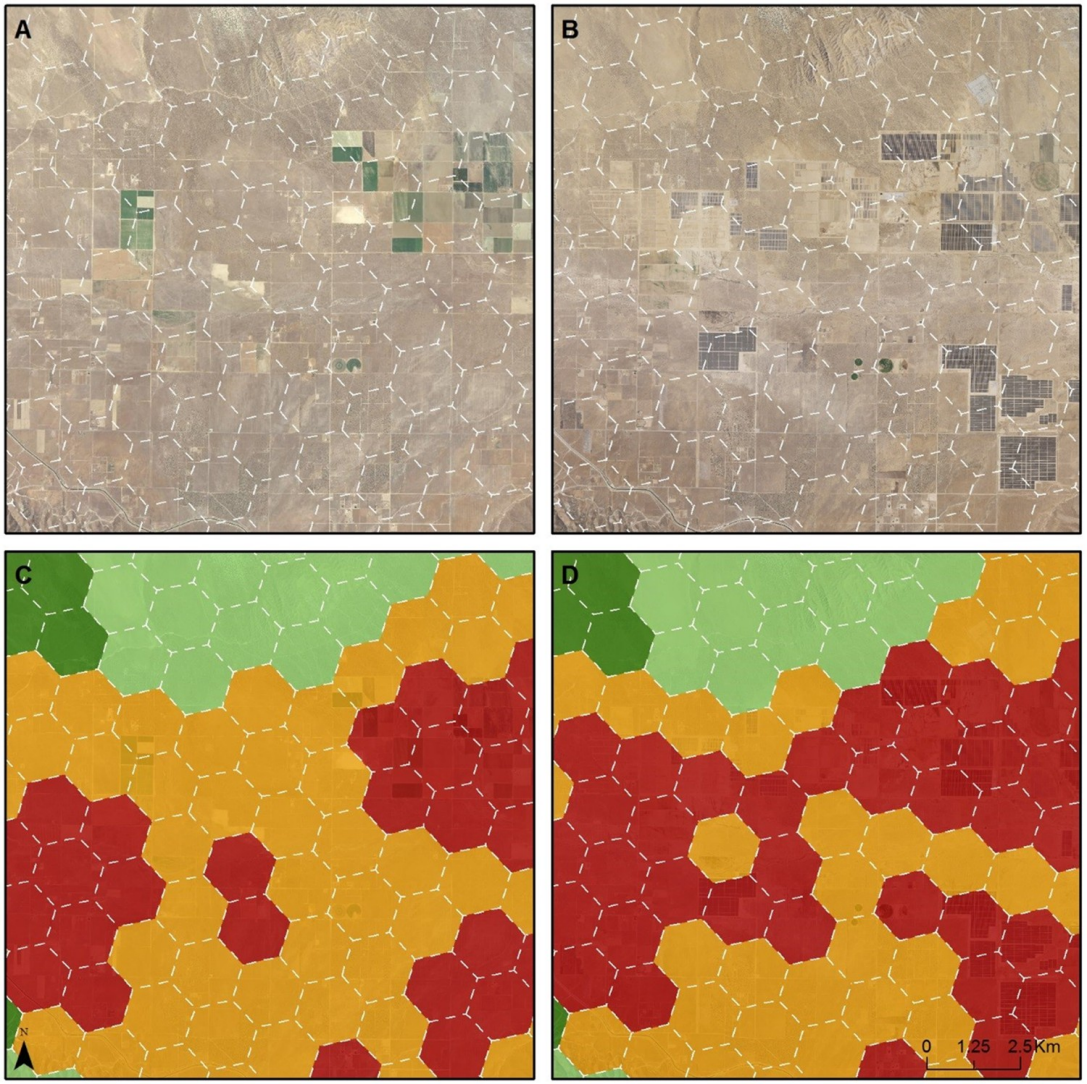
One example of change in conservation value in Western Mojave due to solar energy development. A: 2009 imagery; B: 2016 imagery; C: Conservation Value in 2009; D: Conservation Value in 2017. The four colors shown on maps C and D (dark green, light green, orange, and red) correspond with the conservation value categories defined in Table 2.

**Fig 6:**
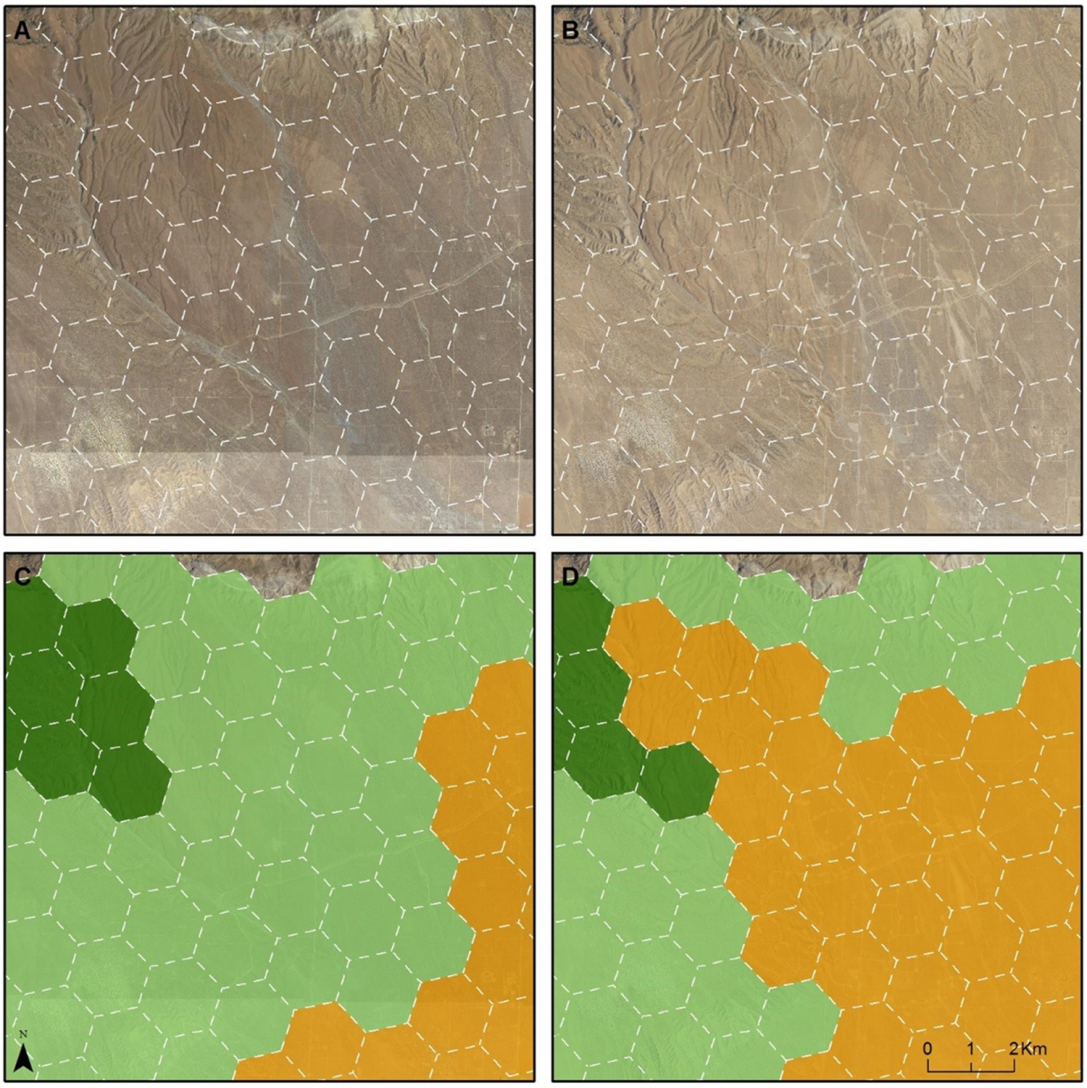
One example of change in conservation value in Western Mojave due to wind energy development. A: 2009 imagery; B: 2016 imagery; C: Conservation Value in 2009; D: Conservation Value in 2017. The four colors shown on maps C and D (dark green, light green, orange, and red) correspond with the conservation value categories defined in Table 2.

The Western Mojave study area is four times the size of the Ivanpah Valley (7,431 vs. 1,870 km^2^, respectively, Table 5 and Fig 2). Compared with their original designation in the MDEA, three of the HPUs were upgraded in conservation value in this analysis. All three upgrades were in the Ivanpah Valley and were due to new tortoise fencing along Interstate 15 that improved the condition of habitat for tortoises within those HPUs. In contrast, the downgrades in this analysis constitute 99 HPUs, equivalent to 256.4 km^2^ of desert lands. Three-quarters (74) of these HPUs were originally designated as Ecologically Core or Ecologically Intact in the MDEA. While the land use and land cover changes driving downgrades were diverse (Table 4), most of the downgrades made to the HPUs (62 out of 99) were due to solar and wind development (Figs 3, 4, 5 and 6).

**Table 5:** Changes in conservation value assignment.

	Total Number of HPUs^1^	HPUs Unchanged	HPUs Changed	HPUs Upgraded	HPUs Downgraded	% Unchanged	% Changed	% Upgraded	% Downgraded	Km^2^ Impacted by Solar Development
Ivanpah Valley- Total	722	696	26	3	23	96.4%	3.6%	0.4%	3.2%	25.81
Ivanpah Valley- Ecologically Core	243	227	16	0	16	93.4%	6.6%	0%	6.6%	18.29
Ivanpah Valley- Ecologically Intact	378	372	6	0	6	98.4%	1.6%	0%	1.6%	6.49
Ivanpah Valley- Moderately Degraded	95	91	4	3	1	95.8%	4.2%	3.2%	1.1%	0.80
Ivanpah Valley- Highly Converted	6	6	0	0	0	100%	0%	0%	0%	0.23
Western Mojave—Total	2,869	2,793	76	0	76	97.4%	2.7%	0%	2.7%	60.99
Western Mojave—Ecologically Core	783	769	14	0	14	98.2%	1.8%	0%	1.8%	0.15
Western Mojave—Ecologically Intact	711	673	38	0	38	94.7%	5.3%	0%	5.3%	7.84
Western Mojave—Moderately Degraded	922	898	24	0	24	97.4%	2.6%	0%	2.6%	25.99
Western Mojave—Highly Converted	453	453	0	0	0	100%	0%	0%	0%	27.09

Land area impacted by solar development in the Ivanpah Valley and the Western Mojave is also shown.

^1^ HPU: hexagonally-shaped planning unit, measuring approximately 2.59 km^2^ (equivalent to one square mile).

There were 23 HPUs (equivalent to 3.2% of the study area) downgraded within the Ivanpah Valley (Figs 2, 3 and 4), whereas there were 76 HPUs (equivalent to 2.6% of the study area) downgraded within the Western Mojave (Table 5). The Ecologically Core and Intact lands that were downgraded to lower categories of conservation value constituted 22 HPUs in Ivanpah Valley, and 52 HPUs in the Western Mojave. In the Ivanpah Valley, most of the downgrades (16 out of 23) occurred on lands that had been designated as the highest conservation value (Ecologically Core) during the MDEA. In contrast, the largest number of downgrades in the Western Mojave (38 of 76) occurred on lands that were assigned as Ecologically Intact in the MDEA. Many of the downgrades (30 of 76) in the Western Mojave were due to wind development (Fig 6); 20 were due to new wind projects, and 10 were due to an expansion of wind development contiguous with an existing wind farm.

In the Ivanpah Valley, 243 of 722 HPUs (33.7%) were categorized as Ecologically Core, 378 (52.4%) as Ecologically Intact, 95 (13.6%) as Moderately Degraded, and 6 (0.8%) as Highly Converted in the MDEA. Of the 23 downgrades that occurred in this analysis, 16 (69.6%) occurred on Ecologically Core lands, 6 (26.1%) on Ecologically Intact lands, and 4 (17.4%) on Moderately Degraded lands. In contrast, in the Western Mojave, 783 of 2,869 HPUs (27.3%) were categorized as Ecologically Core, 711 (24.8%) as Ecologically Intact, 922 (32.1%) as Moderately Degraded, and 453 (15.8%) as Highly Converted in the MDEA. Of the 76 downgrades that occurred, 14 (18.4%) occurred on Ecologically Core lands, 38 (50.0%) on Ecologically Intact lands, and 24 (31.6%) on Moderately Degraded lands. As Highly Converted lands represent the lowest conservation value category, no downgrades occurred on these lands in either study area.

Across the Mojave Desert Ecoregion, solar generation contributed a development footprint of 120.19 km^2^. Of this, 95.58 km^2^ was within the California, and 86.79 km^2^ occurred in our study areas: 25.81 km^2^ in the Ivanpah Valley, and 60.99 km^2^ in the Western Mojave (Table 4). All the lands developed for solar energy in the Ivanpah Valley were BLM lands, and most (18.29 km^2^; 71%) of the land impacted by solar development was comprised of lands that had been designated as Ecologically Core in the MDEA. In contrast, all the solar development in the Western Mojave was on privately owned lands, and most of the lands impacted were lands designated as either Moderately Degraded (25.9 km^2^; 42%) or Highly Converted (27.09 km^2^; 44%) in the MDEA.

## Discussion

Our study demonstrates how the conservation value of the Mojave Desert, as determined by our explicit *a priori* rules, has changed between 2009 and 2017 in areas where renewable energy development has been most intense. Changes in conservation value were dominated by downgrades, which were observed in 99 HPUs within the Ivanpah Valley and Western Mojave study areas. We found renewable energy development to be the leading cause of these declines in conservation value. Together, utility scale solar energy and wind development were directly responsible for 63% of the downgrades observed. We also found that solar energy alone had a development footprint of 86.79 km^2^ in our study.

In the Ivanpah Valley, HPUs that were characterized as Ecologically Core by the Mojave Desert Ecoregional Assessment were disproportionately impacted by downgrades; these lands constituted 33.7% of the study area in 2009, but 69.9% of the 2017 downgrades occurred there. In contrast, in the Western Mojave, HPUs that were characterized in 2009 as Ecologically Intact during the Mojave Desert Ecoregional Assessment were disproportionately impacted by downgrades; these lands constituted 24.8% of the study area in 2009, but 50.0% of the 2017 downgrades occurred there. These differences between the two study areas in the conservation value of lands where development took place reflect differences in the incentives driving development, and the technologies deployed in the Western Mojave vs. the Ivanpah Valley. In the Western Mojave, solar development occurred exclusively on privately owned lands, most of which was of lower conservation value. Wind development was also common. In the Ivanpah Valley, all renewable energy development was solar, and all solar development (18.29 km^2^) occurred on BLM lands. Overall, public lands tended to be more intact, and therefore had very high conservation value.

These findings are important because they provide a comprehensive understanding of the impact of renewable energy development in the Mojave Desert of California, a landscape that has high conservation values overall [7, 10], and has recently experienced rapid development of industrial-scale wind and solar facilities [2]. Our results provide a quantitative assessment of how this development has degraded conservation values over a period of less than a decade, prior to the release of a public land use plan to comprehensively guide such development. Our results will inform necessary updates to the MDEA, which characterized the distribution of conservation values across the entire Mojave Desert Ecoregion using a well-established ecoregional planning approach that set quantitative goals for a suite of conservation targets (521 species, 44 ecological systems, and seeps and springs). With changes in the conservation value of individual HPUs come changes in our ability to meet these conservation goals. This is of concern where Ecologically Core and Ecologically Intact lands have been downgraded to the Moderately Degraded and Highly Converted categories. Too many downgrades, and the ability to meet conservation goals can quickly become a very difficult prospect–at the very least necessitating a reevaluation of landscapes where those goals can be met.

Our methodological approach has limitations. First, these analyses do not provide an understanding of how many km^2^ of habitat have been degraded in ecological function due to factors not visible via satellite imagery such as weed proliferation, wildfires, or other surface disturbances not as obvious but perhaps just as altering to ecological function. Second, our analysis describes changes in conservation value at the scale of the 2.59 km^2^-HPU. Downgrades in HPUs occurred both due to adjacency and due to new disturbance (see Table 2 for decision rules about the amount of disturbance necessary to trigger downgrades, and Table 3 for a summary of why downgrades occurred). Only 25% of an HPU need be disturbed to result in a downgrade in conservation value. Finally, we generated a spatial footprint only for solar development, but not for any other type of land use change, because our intention was to identify the impact of renewable energy development on the conservation values of the Mojave Desert. Our results demonstrate that across our two study areas, 86.79 km^2^ of lands have been converted by this specific type of development.

By comparing our newly generated conservation values map with that released in the 2010 MDEA (Figs 2, 3, 4 and 5), we demonstrate how pre-DRECP legacy projects resulted in a decline in conservation values in the regions of the California Mojave Desert where renewable energy has been developed most rapidly. Our results corroborate at the regional scale the phenomenon of energy sprawl projected through 2040 at the national scale [31]. While solar and wind energy produce only a small fraction of the total electricity across the country, renewable energy facilities cover more land than any other type of electricity production in southern California, and their impact is therefore regionally important.

Renewable energy development is an important global land use change driver [32]. As society shifts from primarily using fossil fuels to harnessing more renewable sources of energy, the demand for land may increase due to the relatively larger areal requirement of solar and wind technologies [33]. In landscapes where renewable energy installations are proposed across large areas, conflicts may arise between energy production and other land uses, such as conservation. Various decision-making tools and methods have been developed to address this challenge by assess and prioritize land use where multiple potential uses compete. Some of these plans, methods, and tools have specifically focused on planning related to renewable energy production [34, 35], and have even included the development of an offset siting method in the Mojave Desert [36] and a least conflict analysis for renewable energy development in the Western subregion of the Mojave Desert in particular [19]. In contrast to these pre-development or pre-land-use-change planning exercises, our study provides a post-development analysis, and quantifies how the actual installation of renewable energy facilities manifested as observable land use change caused a decline in conservation values in the Mojave Desert.

The renewable energy development that took place in the Mojave Desert of California between 2009 and 2017 constitutes the legacy of utility scale solar development and wind facility siting that occurred after enabling conditions spurred development, and prior to the release of the DRECP LUPA. Development patterns may shift with the DRECP LUPA in place, though potential changes to BLM’s land use plan for this area announced in early 2018 introduce uncertainty for both developers and for conservation. Once another decade elapses, we can again reassess the impacts of development on conservation values and determine whether there have been changes in the pace and scale of the decline in conservation values that we observed prior to the completion of the DRECP. This is one potential metric of conservation success for the DRECP, and may serve as a bellwether for regional conservation and development planning in the American West, and beyond.
